# Tuberculosis in the Zoological Gardens

**Published:** 1901-09-21

**Authors:** 


### TUBERCULOSIS IN THE ZOOLOGICAL GARDENS.

Dr. Woods Hutchinson,[1] Professor of Comparative Pathology, University of Buffalo, places on record a series of observations made by him in the post-mortem room of the London Zoological Gardens during the winter and spring of 1898-99, the object being to ascertain whether any light could be thrown upon the predisposing causes of tuberculosis from a study of the incidence of the disease upon the various classes of animals there confined. The three great predispos ng factors which were specially considered were race, food, and housing, and the death records of the gardens for the past five years were considered in conjunction with Dr. Hutchinson's own observations. Some very curious points came out, but whenever an attempt was made to arrive at a wide generalisation some puzzling exception always cropped up, sufficient to show that we have not yet quite got to the heart of the matter. Take the mammals. The highest order, the primates, is by far the most liable to tuberculosis, while next to it comes the lowest division, the marsupials. Ungulates and carnivora, which are usually ranked as on about the same level of specialisation, are widely separated, the former having six times the death-rate of the latter. Within the class of ungulates itself again the most striking contrasts occur, the odd-toed animals—even horses and asses under domestication—being almost completely exempt, while the even-toed ones are extremely liable. Hence a classification according to race tells us nothing. When we come to food and food habits we seem at first on surer ground, the figures pointing strongly towards a greatly increased susceptibility to the disease among vegetable feeders, and a much reduced liability among meat eaters, both mammals and birds. This corresponds closely with the results of veterinary experience, according to which cattle, rabbits, and guinea-pigs are highly susceptible; pigs much less so, and dogs, cats and rats, almost immune. Here, again, however, there are serious exceptions to the apparent rule; the horse, the ass—indeed all the odd-toed ungulates—the goat and the sheep, all pure vegetable feeders, having marked resisting powers and very seldom becoming infected. Evidently although food-habits coincide more nearly with the distribution of tubercle than do racial divisions, yet there is some other factor involved. On the question of housing and artificial heat it was impossible to say much, for on the one hand the animals shut up in warmed houses are mostly tropical animals which would probably die more quickly still out of doors, while on the other hand it is to be noted that some of the older buildings and sheds are worse ventilated and lighted than the modern-heated houses; so that even out-door animals may be under conditions as far from ideal as indoor ones. Certain observations among the monkeys, however, go to enforce very strongly modern ideas regarding the advantages of air-space and light. Coming back then to the apparent exceptions to the general rule as to the immunity of the carnivora, Dr. Hutchinson inquires whether it is the nature of the food, or the habits of life involved in obtaining a carnivorous diet, which is at the root of the matter. That a carnivorous method of life in the majority of instances demands a higher degree of activity, speed, and endurance than a herbiverous one needs little demonstration, and he concludes that where from any cause this higher degree of vitality and dash has been acquired by herbivora then immunity from tuberculosis follows. As then the degree of this vigour and activity may be roughly indicated by the size of the heart relative to the body weight, Dr. Hutchinson has arranged his animals according to this ratio, and certainly there is much in his tables to confirm the view suggested. As confirmation of this view it is to be remembered that size and vigour of the heart have been held by more than one authority to be an important element in the predisposition to tuberculosis among men. Taken altogether, the conclusion arrived at from this preliminary survey of the question of the distribution of tubercle is that it is a matter of vigour, endurance, and resisting power, rather than of race, food, or exposure to infection, and that as these are usually higher in flesh-eaters than in vegetable feeders, the former possess a marked relative immunity.

[1] New York Med. Rec., Aug. 25.

